# Predicting neonatal RDS with fetal pulmonary artery doppler: a diagnostic performance and ROC curve analysis

**DOI:** 10.1186/s13089-025-00456-y

**Published:** 2025-11-05

**Authors:** Ishan Kumar, Karan Kukreja, Ashok Kumar, Priyanka Aggarwal, Shikha Sachan, Ashish Verma

**Affiliations:** Banaras Hindu University, Varanasi, India

**Keywords:** Respiratory distress syndrome, Newborn, Doppler ultrasonography, Pulmonary artery, Prenatal diagnosis

## Abstract

**Study design:**

Prospective observational diagnostic accuracy study.

**Methods:**

This study evaluated Doppler parameters of the fetal main pulmonary artery (MPA) as potential non-invasive predictors of RDS and to assess their relationship with gestational age and postnatal respiratory outcomes. Pulsed-wave Doppler of the fetal MPA was performed and Doppler indices—pulsatility index (PI), resistance index (RI), peak systolic velocity (PSV), and acceleration time/ejection time ratio (At/Et)—were recorded over three cardiac cycles and averaged. Values were compared after delivery between neonates with and without RDS.

**Results:**

In normally developing fetuses, PI and RI decreased while At/Et increased with advancing gestational age. In contrast, fetuses who developed RDS showed persistently low At/Et and elevated PI and RI values. Cutoff values of At/Et < 0.24, RI > 0.8, and PI > 2.16 were effective in predicting RDS. The severity of RDS was associated with greater deviations from these values. Altered Doppler indices were found to be independent predictors of RDS in preterm neonates.

**Conclusion:**

Fetal MPA Doppler parameters serve as reliable, non-invasive predictors of RDS and the severity of RDS in preterm neonates.

## Introduction

Respiratory distress syndrome (RDS) is the most common cause of respiratory failure in neonates, primarily resulting from a deficiency in pulmonary surfactant. It predominantly affects premature neonates, though it can occasionally occur in full-term infants. The diagnosis of fetal lung maturity may be assessed through amniocentesis, an invasive procedure, following which the amniotic fluid sample is tested for the lecithin/sphingomyelin (L/S) ratio, the presence or absence of phosphatidylglycerol, and the lamellar body count. However, due to the invasive nature and associated risks of amniocentesis, there is a growing need for reliable non-invasive alternatives, which would be a more acceptable screening tool [1, 2].

**Fig. 1:**
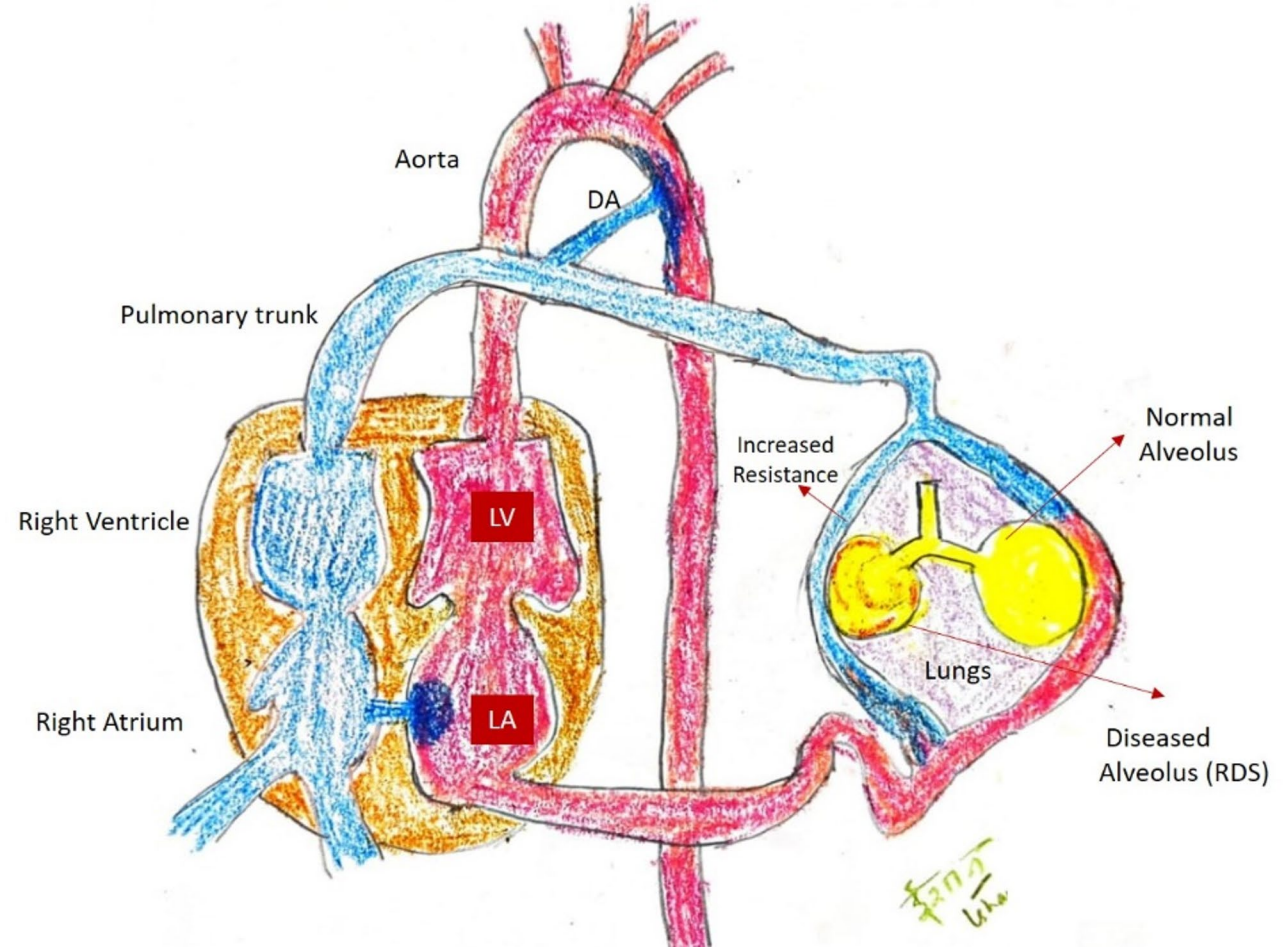
Diagram showing the basis of vascular changes in neonatal respiratory distress syndrome

Lung maturation is closely linked to pulmonary vascular changes, including decreased resistance in the pulmonary arterial system, increased elasticity and caliber of the pulmonary arteries, and angiogenesis. As the lungs mature, the growing lung tissue reduces lung pressure. This vascular remodeling process is crucial for proper lung development and function. In neonates with RDS, however, this normal vascular remodeling is impaired (Fig. 1), contributing to the pathophysiology of the condition [3, 4]. This can form a basis for identifying potential biomarkers to anticipate the condition. As a non-invasive modality, fetal MPA Doppler has the potential to serve as an index test for assessing the risk of neonatal RDS, particularly in pregnancies at risk of preterm delivery.

This study aims to assess the diagnostic performance of fetal MPA Doppler parameters for predicting neonatal RDS, with the goal of establishing their clinical utility as a non-invasive screening tool.

## Materials and methods

### Study design

This was a prospective observational study and was conducted at a University-based tertiary care hospital over a 2-year period. This was approved by Institute ethics committee and informed consent was taken from the parents of all the neonates included in this study.

### Participants

Participants were identified from those referred for routine fetal ultrasound assessments in the third trimester. A consecutive sampling method was used, including all eligible patients referred for third-trimester fetal ultrasound during the study period. Inclusion criteria was singleton pregnancies with more than 28 weeks of gestation, referred from fetal Ultrasound. The exclusion criteria was (i) Fetus with structural/suspected chromosomal anomalies, (ii) multifetal pregnancies (iii) maternal comorbidities including chronic diabetes, gestational diabetes, or preeclampsia (iv) patients lost to follow up or delivered at outside Institute.

### Test methods

**Fig. 2:**
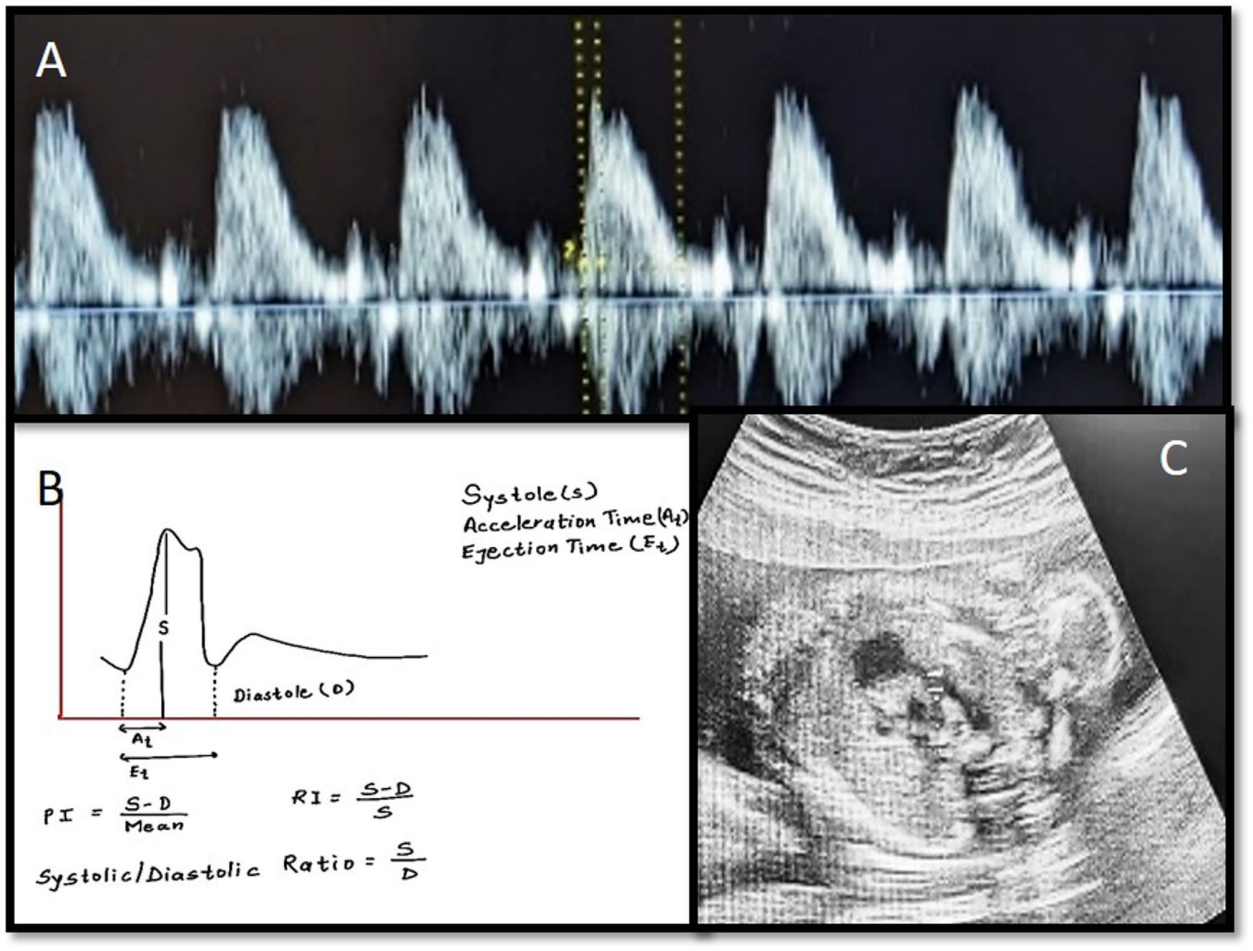
**a** Pulmonary artery wave form with classical spike (first peak) and gradual declining slope (spike and dome pattern). **b** Calculation of Doppler values **c**) sample ate is placed at mid pulmonary trunk

Index test: Ultrasound and doppler study were performed by a single operator (IK) with 10 years of experience. Routine biometry, including measurements of fetal growth parameters, was performed as part of the standard obstetric ultrasound examination. Standard Cardiac assessments were conducted including four-chamber view, the outflow tracts, and the three-vessel view of the fetal heart. The main pulmonary artery (MPA) was followed in axial view and pulsed wave sample was kept at mid part of pulmonary artery. A sample gate width of 2–4 mm was used and the angle of insonation was kept below 20 degrees. Pulmonary artery was identified with a classical “spike and dome” pattern. (Fig. 2) Care was taken to distinguish from ductus arteriosus which usually has a higher peak velocity, triangular shape (dome is missing). Doppler measurements were obtained from three consecutive cardiac cycles, and the average value was calculated to minimize any variability. Pulsatility index (PI), resistance index (RI), peak systolic velocity (PSV) and systolic/diastolic ratio (S/D ratio) were charted for each measurement.

Reference standard : All the fetuses were followed up till the delivery. Those born at 37 weeks or higher were considered “term” birth and rest were considered as preterm pregnancy. Neonatal care was done under one of the authors of this study who was blinded to the findings of Doppler study (AK). Maternal and fetal parameters were noted at birth including birth weight, APGAR score (at 1 and 5 min). RDS was diagnosed when neonates exhibited tachypnea, grunting, nasal flaring, and retractions. In addition, the requirement for supplemental oxygen of 0.4 FiO2 or higher for at least 24 h was an essential criterion. Chest X-rays showing the characteristic features of RDS, such as diffuse bilateral infiltrates and a ground-glass appearance, confirmed the diagnosis. Silverman Anderson score (0–10) was used to assess the severity of RDS with score 0 = no distress; 1–3: mild distress; 4–7: moderate distress; 7–10: severe distress [5]. Neonates diagnosed with neonatal acute respiratory distress syndrome (NARDS) were excluded from the study, in accordance with the Montreux Consensus Definition criteria i.e. those with acute onset of respiratory failure following a known clinical insult.

### Analysis

Statistical analyses were performed using Jamovi software (The Jamovi project [2019]. jamovi. [version 1.1.9] [computer software]; https://www.jamovi.org). Comparisons between groups (RDS vs. non-RDS, and among RDS severity groups) were performed using the independent samples *t*-test or one-way ANOVA. Chi-square test was used for categorical variables. Receiver Operating Characteristic (ROC) curve analysis was conducted to determine the predictive performance of fetal pulmonary artery Doppler indices in identifying neonates at risk for RDS. Cutoff values for Doppler parameters were determined post hoc using ROC analysis and Youden’s Index, and were considered exploratory. A linear regression model was used to assess the association of Doppler indices (AT/ET, RI, PI), gestational age at delivery, and 5-min APGAR score with the severity of respiratory distress, as measured by the Silverman-Anderson score. Variables with *p* < 0.05 were considered statistically significant throughout the analysis.

## Results

### Participants

**Fig. 3:**
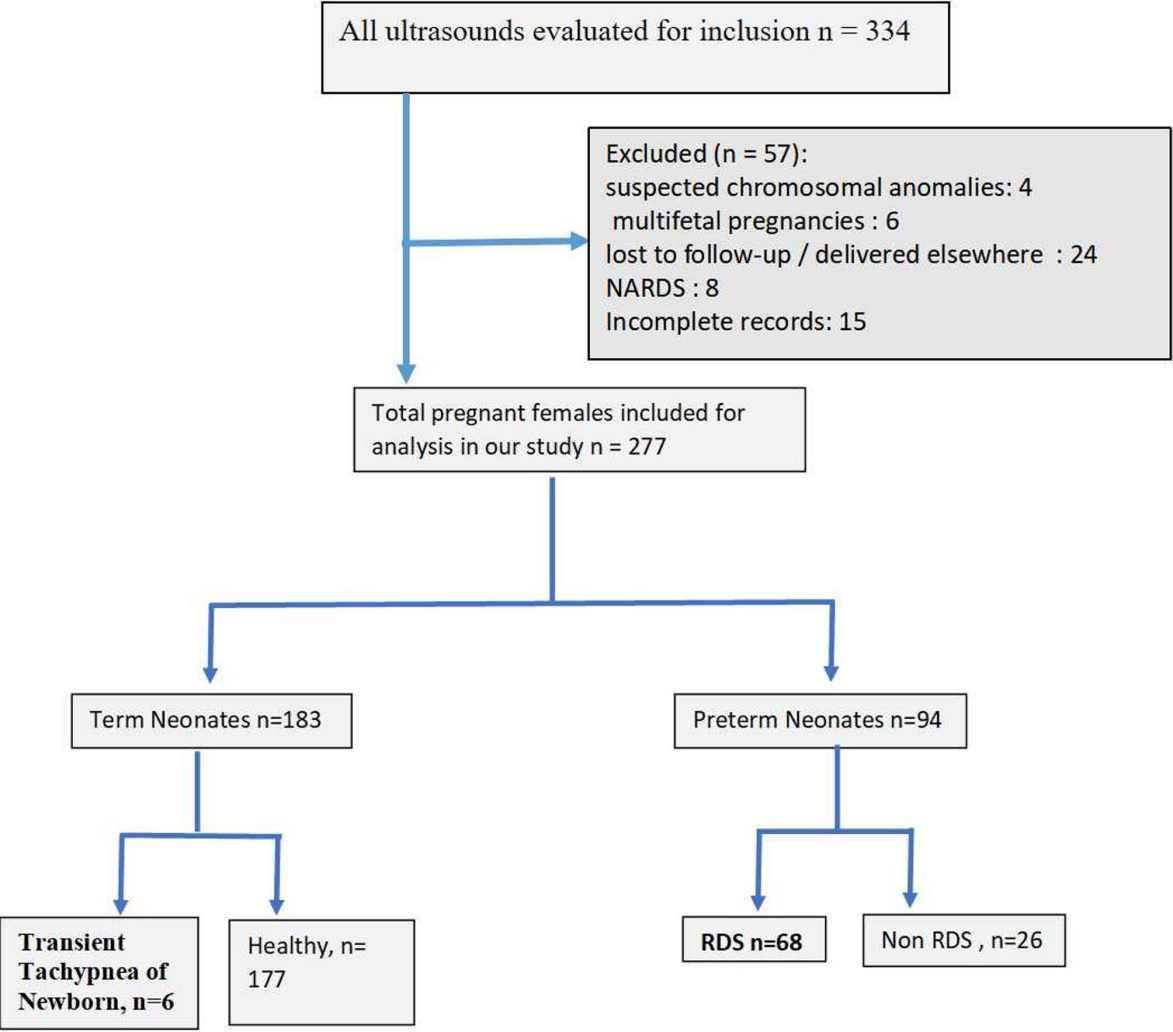
Flowchart depicting included patients in this study

A total 334 patients were selected for ultrasound out of which 57 were excluded and 277 patients were included in this study (Fig. 3). The mean maternal age was 27.4 ± 4.1 years, with a range from 23 to 39 years. Table 1 summarizes the, birth weight, APGAR score and other characteristic of the cohort. Of the 277 neonates, 183 delivered at 37 weeks or later (term) while 94 were preterm deliveries. Among the neonates who did not develop RDS, 6 term neonates were diagnosed with transient tachypnea of the newborn (TTN); the remaining neonates had no signs of respiratory distress. In the preterm group, 68 neonates were diagnosed as RDS (Fig. 3). Neonates diagnosed with neonatal acute respiratory distress syndrome (NARDS) were excluded from the study, in accordance with the Montreux Consensus Definition criteria i.e. those with acute onset of respiratory failure following a known clinical insult.

**Table 1:** Basic characteristic of the cohort

Parameter	Mean value (range)
Maternal age (years)	27.4 ± 4.1 (23 to 39 years)
Gestational age (weeks)	33.8 ± 3.5 (29 to 39 weeks)
APGAR score at 1 min	7.5 ± 1.2 (4–8)
APGAR score at 5 min	8.5 ± 1.0 (6–9)
Birth weight (kg)	2.75 ± 0.45 (2.0–3.5)
Parity distribution	Nullipara : 199 ; Multipara: 78
Neonatal sex distribution	Female: 147; Male: 130

### Variation with increasing gestational age

**Fig. 4:**
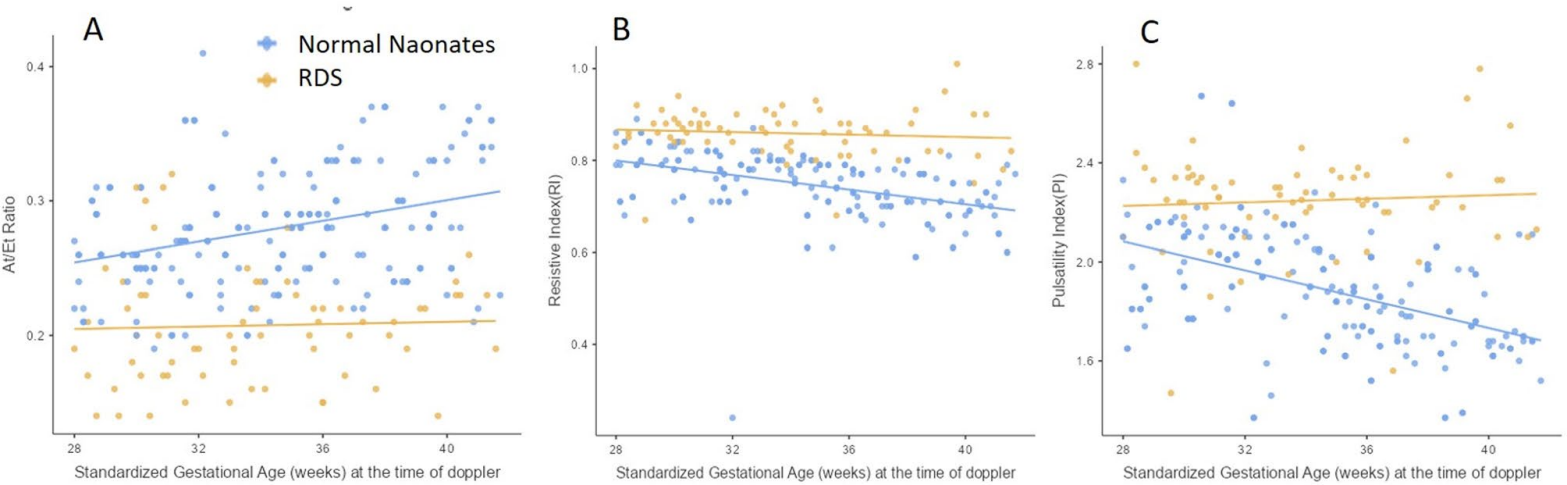
**a** Scatter plot showing variation of the AT/ET ratio, **b** resistive index and **c** pulsatility index, with gestational age in fetuses who developed RDS (yellow) and who did not (blue)

In the neonates who did not develop RDS, the AT/ET ratio showed a mild increasing trend with increasing gestational age (28 weeks onwards), while in the RDS group, it did not increase and showed lower value. The resistive index (RI) the pulsatility index (PI) demonstrated a slight decreasing trend in the healthy neonates, while it remained relatively stable in the RDS group (Fig. 4).

### Comparison between the RDS and non-RDS groups

The AT/ET ratio was lower in the RDS group (0.21 ± 0.04) compared to the non-RDS group (0.28 ± 0.04, *p* = 0.001). The Resistive Index (RI) and Pulsatility Index (PI) were significantly higher in the RDS group (RI: 0.86 ± 0.05, PI: 2.25 ± 0.21) compared to the non-RDS group (RI: 0.75 ± 0.07, PI: 1.89 ± 0.23). No significant differences were observed in Peak Systolic Velocity or S/D Ratio (Table 2).

**Table 2:** Comparison between respiratory distress syndrome (RDS) group and Non RDS group

	Parameter	RDS group (*n* = 68)	Non-RDS group (*n* = 209)	*p*-value
Doppler parameters	At/Et ratio	0.21 ± 0.04	0.28 ± 0.04	0.001
	Resistive index (RI)	0.86 ± 0.05	0.75 ± 0.07	0.003
	Pulsatility index (PI)	2.25 ± 0.21	1.89 ± 0.23	0.002
	Peak systolic velocity	59.82 ± 13.02	60.69 ± 10.99	0.588
	S/D ratio	4.85 ± 1.41	4.78 ± 1.25	0.66
Neonatal parameters	Neonatal birth weight (kg)	2.24 ± 0.23	2.90 ± 0.36	0.002
	GA at delivery (weeks)	34.54 ± 3.51	38.45 ± 2.33	0.04
	APGAR 1 min	Median: 5.0	Median: 7.0	0.39
	APGAR 5 min	Median: 7.0	Median: 9.0	0.33
Maternal parameters	Maternal age	25.51 ± 3.36	26.16 ± 3.91	0.224
	GA at the time of Doppler	33.85 ± 3.73	34.45 ± 3.76	0.26

Table provides the comparison of various Doppler, maternal and neonatal parameters between RDS group and non-RDS group

**Fig. 5:**
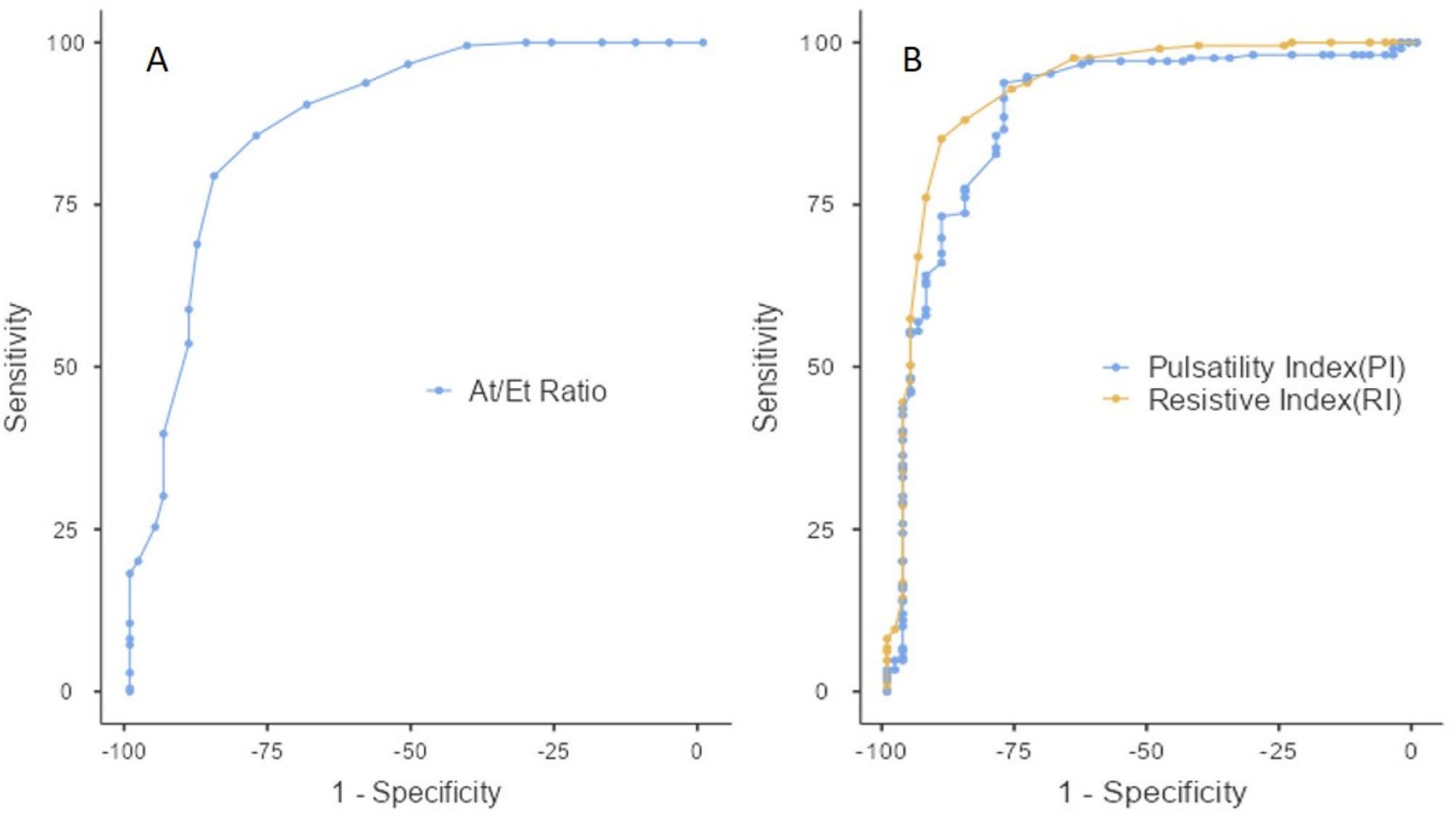
Receiver operated characteristic curve (ROC) to predict the development of neonatal RDS for **a** pulsatility and resistive indices and **b**) AT/T ratio

Neonates in the RDS group had lower birth weights and earlier gestational ages at delivery (compared to the non-RDS group. No significant differences were found in APGAR scores at 1 and 5 min. Maternal age and gestational age at the time of Doppler examination were similar between both groups. Receiver operated characteristic(Fig. 5) curve (ROC)- derived cutoff values—At/Et ratio of 0.24, resistance index (RI) of 0.8, and pulsatility index (PI) of 2.16—could predict the risk of RDS with high accuracy (Table 3).

**Table 3:** Receiver operated characteristic curve (ROC)- derived cutoff values

Parameter	Cut-off	Sensitivity (%)	Specificity (%)	PPV (%)	NPV (%)	Youden index	AUC
Resistive index	o.8	85.17	89.71	96.22	66.3	0.749	0.926
Pulsatility index	2.16	93.78	77.94	92.89	80.3	0.717	0.894
AT/ET ratio	0.24	85.65	77.94	92.27	63.86	0.636	0.881

Table provides the ROC- dertived cut-off and their accuracy for Doppler parameters in predicting RDS

Among the control group, a subgroup of neonates diagnosed with TTN (*n* = 6) was analyzed separately to assess comparability with rest of the healthy controls. The mean AT/ET ratio, RI, PI, PSV and S/D ratio in TTN cases were 0.240 ± 0.41, 0.750 ± 0.06, 1.88 ± 0.18, 55.3 ± 9.27 cm/s, and 5.68 ± 1.05 respectively, compared to 0.28 ± 0.04, 0.748 ± 0.07, 1.90 ± 0.23, 60.9 ± 11.0 cm/s, and 4.75 ± 1.24 in rest of the healthy controls. None of these differences reached statistical significance, confirming that TTN cases exhibited similar pulmonary hemodynamics to healthy neonates.

### Association with increasing RDS severity

**Fig. 6:**
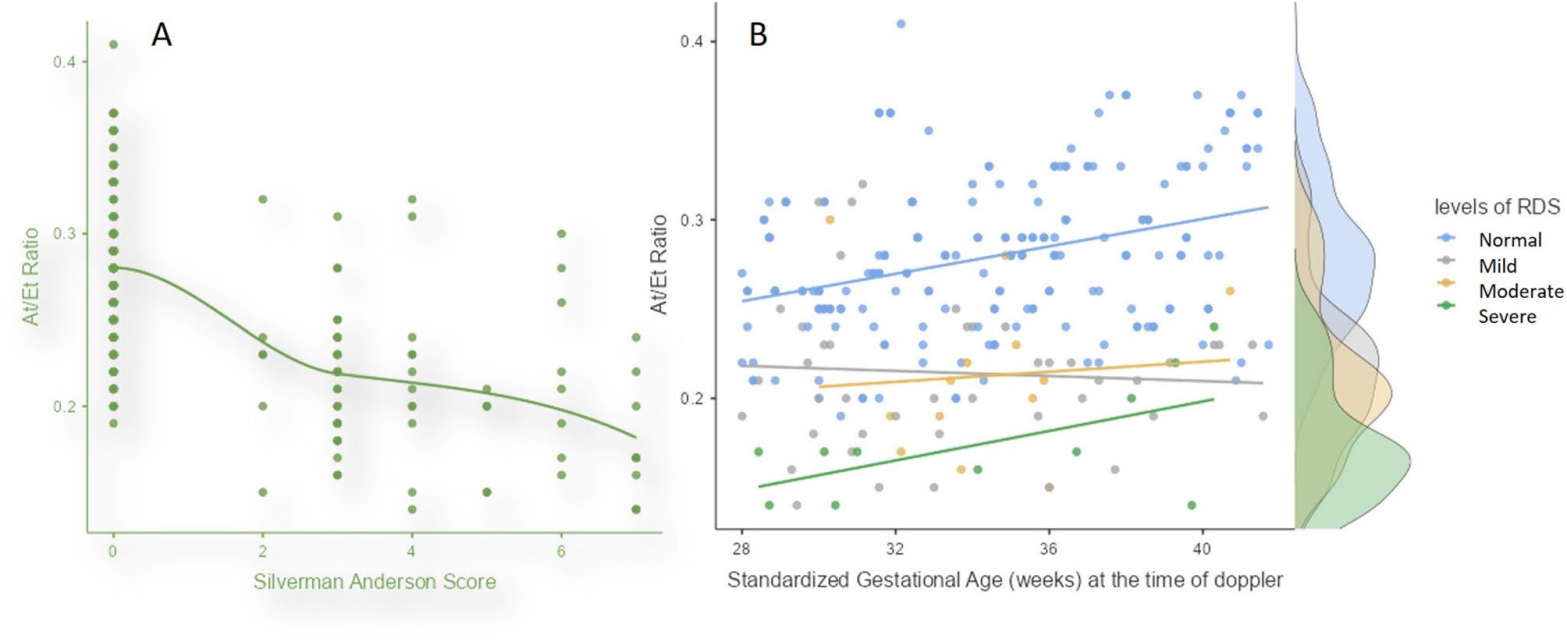
**a** Scatter plot between AT/ET ratio and Anderson Silverman score. **b** Scatter plot showing variation of the AT/ET ratio, with increasing gestational age in fetuses who developed mild (grey), moderate (yellow) and severe (green) RDS as well as normal fetuses (blue)

The AT/ET ratio progressively decreased from the healthy group (0.28 ± 0.04) to the mild, moderate and severe RDS group (0.17 ± 0.03). Similarly, the RI and PI significantly increased with the severity of RDS (Table 4). No significant difference was observed in the Peak Systolic Velocity (PSV) and S/D ratio across the groups. Scatter plot showed inverse correlation between the AT/ET ratio and Silverman Anderson score (Fig. 6). With increasing gestational age, severe RDS group showed a mildly increasing value of A/ET ratio, unlike mild and moderate RDS which showed a relatively constant value (Fig. 6). The linear regression model was used to predict the Silverman-Anderson score using fetal pulmonary artery Doppler indices, gestational age at delivery, and the 5-min APGAR score. The model explained 60% of the variance in the Silverman-Anderson score (R² = 0.600). The At/Et ratio, RI, PI, and gestational age were statistically significant, with the At/Et ratio having a strong negative effect, and the others showing positive correlations with the score. The 5-min APGAR score, however, was not significant.

**Table 4:** Association with RDS severity

Parameters	Normal	Mild RDS (*n* = 43)	Moderate RDS (*n* = 14)	Severe RDS (*n* = 11)	*P* value
At/Et	0.28 ± 0.04	0.22 ± 0.04	0.21 ± 0.04	0.17 ± 0.03	< 0.001
RI	0.75 ± 0.07	0.84 ± 0.05	0.86 ± 0.06	0.9 ± 0.05	< 0.001
PI	1.9 ± 0.23	2.2 ± 0.21	2.3 ± 0.17	2.4 ± 018	< 0.001
PSV (cm/s)	60.6 ± 10.99	59.95 ± 12.8	62.73 ± 15.4	55.6 ± 10.2	0.44
S/D Ratio	4.77 ± 1.24	4.85 ± 1.36	4.6 ± 1.5	5.18 ± 1.5	0.8

Comparison of mean values of Doppler parameters between normal (Non RDS), mild moderate and severe RDS neonates

### Comparison within preterm group (n = 94)

Comparing preterm RDS neonates (*n* = 68) to preterm healthy neonates (*n* = 26), AT/ET ratio was lower in the preterm RDS group (0.21 ± 0.04) compared to the healthy preterm neonates (0.28 ± 0.04). Also, RI and PI were significantly higher in the preterm RDS group (Table 5). No significant difference was found in PSV and S/D ratio between the two groups.

**Table 5:** Comparison in preterm neonates

Variable	Preterm RDS neonates mean (SD)	Preterm healthy neonates mean (SD)	*P*-value
Total count	68	26	0.40
Standardized GA at delivery (weeks)	34.54 (3.51)	34.27 (1.57)	0.616
Neonatal birth weight (kg)	**2.02 (0.21)**	**2.45 (0.13)**	**0.034**
At/Et ratio	**0.21 (0.04)**	**0.28 (0.04)**	**0.028**
Resistive index (RI)	**0.86 (0.05)**	**0.77 (0.05)**	**0.045**
Pulsatility index (PI)	**2.25 (0.21)**	**1.91 (0.18)**	**0.037**
PSV	59.82 (13.02)	60.13 (11.88)	0.9153
S/D ratio	4.85 (1.41)	5.26 (1.21)	0.1806

Comparison of the various Doppler and clinical parameters within the pre-term group (*n* = 94) between those who developed RDS and those who did not

## Discussion

The results of this study shows that Doppler of pulmonary artery can be utilized to predict the impending RDS in a neonate. AT/ET ratio lower than 0.24, and RI and PI higher than 0.8, and 2.16 have been shown to predict RDS with high accuracy. Furthermore, increasing severity of RDS is associated with lower AT/ET ratios and higher RI and PI values. In preterm neonates, these altered Doppler parameters serve as independent predictors for the development of RDS, suggesting their potential utility in early identification and management of at-risk infants.

Little is known about the blood flow patterns and their developmental changes in the pulmonary arteries. Chaoui et al. first described that with increasing gestational age (16–40 weeks), there is a lengthening of acceleration time (AT), an increase in the AT/ejection time (AT/ET) ratio, a rise in peak systolic velocity (PSV), and a decrease in pulsatility index (PI), reflecting reduced vascular resistance [6]. In the human fetus, Doppler waveforms from the proximal pulmonary artery branches exhibit a distinct shape i.e. a needle-like systolic peak, rapid acceleration, early deceleration, and a dicrotic notch with occasional reverse flow, unlike those seen in animal studies. Most healthy fetuses demonstrate low diastolic forward flow [7]. St. John Sutton et al. assessed fetal pulmonary perfusion by comparing flow through the pulmonary trunk and ductus arteriosus, revealing a four-fold increase in pulmonary blood flow between 20 and 35 weeks’ gestation [8]. Structurally, the basic branching pattern of the pulmonary arteries forms by 16 weeks, with further arborization decreasing impedance, although not to the extent observed in fetal lambs, where a 40-fold increase in peripheral arteries is reported [3, 9]. Kitabatake et al. showed that shorter AT and reduced AT/ET ratios correlate with higher pulmonary arterial pressures, a relationship supported by later studies [10]. Postnatally, a significant rise in AT and AT/ET ratio occurs after 6 h of life due to the rapid drop in pulmonary vascular resistance [11]. Laudy et al. further explored waveform patterns by evaluating flow velocity profiles at three levels [12]: proximal branches, middle branches (defined as segments equidistant from the outer border of the fetal heart and the inner thoracic wall), and distal branches (the most peripheral pulmonary arterial segments, located near the inner thoracic wall). They found good repeatability (CV < 15%) and observed that middle branches showed waveforms similar to proximal ones, with gestational age-related changes in diastolic velocity, PI, and systolic/diastolic ratios. These waveforms featured a sharp systolic peak followed by a more gradual decline and, in 45% of cases, a brief reverse flow at diastole onset. In contrast, distal branches exhibited a monophasic forward flow throughout the cardiac cycle, with stable velocity parameters except for PI [12, 13].

Our findings are consistent with several studies that have investigated Doppler parameters in relation to RDS in preterm neonates [14–22]. Buke et al. have suggested a similar trend but a higher cut-off value of 0.32 for AT/ET below which RDS can be predicted, although they had a limited number of RDS cases (*n* = 22) in their study [14]. A similar higher cut-off value of 0.305 for At/Et has been suggested by Moety et al. who studied 55 RDS neonates, despite having similar mean AT/ET in the RDS group (0.21) [1]. This higher cut-off, also was suggested by Guan et al., Schenone et al. (0.31), Khalifa et al., and Hassan et al. (0.30), while a lower value of 0.28 has been suggested by Keshuraj et al. These differences could also be attributed to difference in the population studied, which highlights the importance of having a reference value of each population. The suggested cut off value for RI and PI were similar across the studies including the present study. However, two initial studies have reported contrasting results. Azpurua et al. showed an inverse relationship between the AT/ET ratio and the lecithin/sphingomyelin ratio, but this was based on a single RDS case, and Kim et al. observed an inverse relationship between the AT/ET ratio and lung maturity, again limited by 11 RDS cases [18, 19].

One of the key strengths of our study is the relatively large sample size of 68 RDS patients, which is the largest data till date for analyzing the relationship between Doppler parameters and RDS. Additionally, to the best of our knowledge, our study is the first to demonstrate a clear association between altered Doppler values and the severity of RDS measured by the Silverman score. Also, we could demonstrate the differences in the trend of the Doppler values with progressing gestational age between RDS and healthy fetuses.

However, there are some limitations to consider. First, we did not assess interobserver agreement, as all the Doppler was performed by a single observer. As the waveform of MPA can present with different appearances at different gestational age, it is important to assess interobserver variation of measurement of the indices, especially, AT/ET ratio. Additionally, our study excluded fetuses < 28 weeks of gestational age, precluding the assessment of early (2nd trimester) trend in pulmonary artery indices. This study did not include high-risk subgroups such as cases with placental insufficiency, preeclampsia, gestational diabetes, or other maternal medical conditions, which may limit the applicability of the findings to these populations. Also, we acknowledge that more comprehensive clinical data, oxygenation parameters at birth, including early respiratory status, progression of symptoms, and timing of clinical events, were not analysed, which may have enriched the interpretation of our results.

In conclusion, this study demonstrates that fetal pulmonary artery Doppler parameters are reliable, non-invasive predictors of RDS in preterm neonates, with abnormal values correlating with both the risk and severity of the condition. Additionally, it establishes reference values for these parameters in normal fetuses, providing a useful baseline for clinical comparison and early risk assessment.

## Data Availability

No datasets were generated or analysed during the current study.
